# Sexually dimorphic tumor suppression by small mitochondrial *Arf*

**DOI:** 10.18632/oncotarget.26651

**Published:** 2019-02-08

**Authors:** Jolieke G. van Oosterwijk, Heather Tillman, Charles J. Sherr

**Affiliations:** ^1^ Department of Tumor Cell Biology, St. Jude Children's Research Hospital, Memphis, TN, USA; ^2^ Department of Pathology, St. Jude Children's Research Hospital, Memphis, TN, USA; ^3^ Howard Hughes Medical Institute, St. Jude Children's Research Hospital, Memphis, TN, USA

**Keywords:** Arf tumor suppressor, small mitochondrial Arf (smArf), smArf sexual dimorphism

## Abstract

Internal translational initiation of the mRNA encoding the Arf tumor suppressor yields an N-terminally truncated small Arf protein (smArf) that lacks amino acid residues required for Mdm2 binding and p53 activation. Here, we report that female, but not male, mice engineered to produce only smArf in lieu of the full-length Arf protein retain residual, sexually dimorphic tumor suppressive activity.

The alternative reading frame protein (mouse p19^Arf^; human p14^ARF^) encoded by the *Cdkn2A* (*Ink4a/Arf*) tumor suppressor complex inhibits the Mdm2 E3 ligase to activate p53. A conserved in-frame methionine codon (M45 and M48 in mouse and human *Arf* mRNAs, respectively) initiates translation to produce a truncated, unstable small mitochondrial polypeptide (smArf) in addition to the nuclear full-length, protein [1]. Although the mouse p15^smArf^ protein lacks amino acid residues required for interacting with Mdm2 and is overtly deficient in triggering p53-dependent tumor suppressor activity, it surprisingly rescues focal developmental defects observed in *Arf*-null mice that synthesize neither p19^Arf^ nor p15^smArf^; conversely, mice engineered to produce only a mutant p19^Arf-M45A^ protein (encoding alanine in lieu of M45) retain p53-dependent tumor suppressor activity but continue to exhibit the fully penetrant developmental ocular and spermatogenesis defects seen in *Arf*-null animals [2].

Syngeneic C57BL/6 mice engineered to produce either p15^smArf^ or p19^ArfM45A^ were sublethally irradiated (IR) as neonates (4 Gy, day 6 postpartum) and observed for tumor development for one-year thereafter. Males of both strains and *Arf*-null females developed tumors with mean latencies of 16-17 weeks, as previously reported for *Arf*-null mice of either sex [3]. In stark contrast, *smArf* females were unexpectedly more resistant to tumor formation (mean latency 38 weeks, *p* < 0.0005) (Figure 1A). Regardless of tumor latencies, an indistinguishable tumor spectrum arose in irradiated *smArf* males and females (62% sarcomas, 38% lymphomas) similar to that observed in *Arf*-null mice [3]. Fewer than 10% of irradiated *Arf*^M45A^ mice (or parental *Arf*^+/+^ mice) developed tumors throughout 60 weeks of observation. Therefore, although the p53-activating N-terminal domain of p19^Arf^ is required for robust tumor suppression, p15^smArf^ retains a residual tumor suppressive function manifested specifically in females. In short, irradiated *smArf* mice exhibit sexually dimorphic tumor suppressor activity.

**Figure 1:**
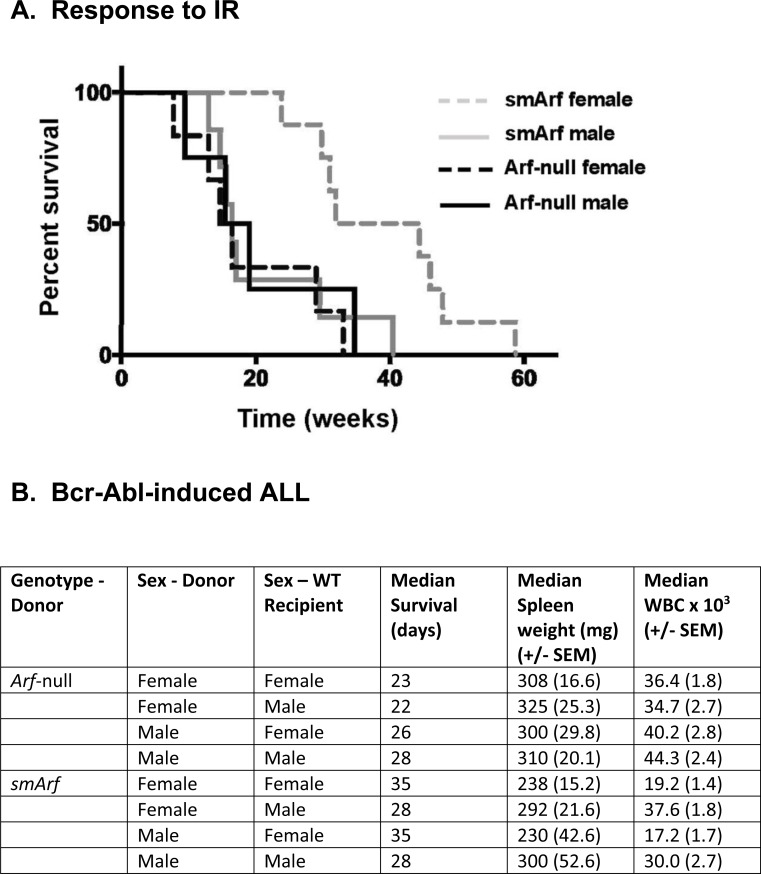
Differential responses of females and males to oncogenic challenge **A.** Survival of neonatally irradiated mice of the indicated genotypes. Comparison of Kaplan-Meier survival curves was determined by Cox regression and Kruskal-Wallis test: smArf females versus any other group (*p* < 0.0005). All moribund animals developed sarcomas or lymphomas regardless of genotype or tumor latency. **B.** Development of acute lymphoblastic leukemia (ALL) after infusion of male or female donor *Arf*-null, Bcr-Abl-positive or *smArf*, Bcr-Abl-positive pro-B cells into syngeneic wild-type (WT, *Arf*^+/+^) recipients of the same or opposite sex. Eight individual cohorts each included 12 recipient WT mice that received 20 cells of the indicated donor genotypes per animal. Spleen weights and white blood cell (WBC) counts were determined at the time of sacrifice. Comparisons of WBC counts and spleen weights were generated using student's two-tailed t test. Donor *smArf*, Bcr-Abl+ cells of either sex yielded significantly reduced spleen weights and lower peripheral WBC counts in female vs male recipients (each *p* < 0.05). No such differences in signs of disease development in recipients were observed following infusion of control *Arf*-null, Bcr-Abl+ donor cells. All statistical analyses were performed using R statistical software version 3.4.2. Ethical use of animals: All animal experiments were performed according to NIH guidelines and Institutional Animal Care and Use Committee-approved protocols.

Like Interleukin-7-dependent *Arf*-null or *Trp53*-null pro-B cells, pro-B cells cultured from *smArf* mice are capable of continuous cytokine-dependent self-renewal. When transduced with retroviral vectors encoding the Bcr-Abl kinase, the IL7-dependence of these cells is abrogated, and they initiate acute lymphoblastic leukemia (ALL) when infused intravenously into unconditioned, syngeneic wild type (WT, *Arf+/+*) recipient mice [2, 4]. Previous calculations of dose-dependent disease onset indicated that populations of genetically marked Bcr-Abl+, *Arf*-null donor cells expand ~10-fold *in vivo* in syngeneic WT animals over 3-day intervals. Under these conditions, healthy C57BL/6 mice receiving only 20 *Arf*-null, Bcr-Abl+ donor pro-B cells succumb to lethal ALL within 30 days of infusion, whereas mice receiving 2 × 10^5^ *Arf*^+/+^, Bcr-Abl+ donor pro-B cells develop no disease [4, 5].

To determine whether the sex of recipient animals might play any role in determining the rate of onset and clinicopathological signs of disease, Bcr-Abl+ pro-B cells from either *Arf*-null or *smArf* male or female donor mice were infused into healthy syngeneic WT recipients of either sex (Figure 1B). No significant differences in tumor development among cohorts were observed when recipient animals were infused with 2,000 male or female donor cells per mouse. However, because of the highly aggressive nature of this ALL model [4, 5], we infused limiting numbers of green fluorescent protein (GFP)-marked leukemia-initiating cells (only 20 per recipient mouse) in an attempt to magnify any differences between eight individual cohorts of 12 mice each (Figure 1B). Control *Arf*-null, Bcr-Abl+ donor cells of either sex still rapidly induced ALL in cohorts of wild-type male or female recipients without discernibly significant sex bias. In contrast, healthy syngeneic female mice receiving either male or female Bcr-Abl+, *smArf* donor cells exhibited an increased median survival of one week compared to male recipients; moreover, females developed less aggressive disease than males as manifested by comparatively reduced median spleen weights and lower peripheral white blood cell (WBC) counts (each *p* < 0.05) at the time of sacrifice (Figure 1B). All moribund mice succumbed to typical Bcr-Abl-induced ALLs marked by progressive lethargy, ruffling of fur, hunched posture, hind limb paralysis, domed heads, seizures, and respiratory distress [5]. Slower ALL progression in female mice tended to correlate with more consistent signs of CNS involvement. At necropsy, we documented hepatosplenomegaly and histological evidence of disseminated GFP+ B-cell infiltration in liver, spleen, lymph nodes, spinal cord, and meninges, as previously reported [5]. Therefore, the rate and severity of ALL development following injection of donor *smArf* leukemia-initiating cells depended on the sex of the recipient hosts with female mice exhibiting greater resistance to challenge by *smArf* donor cells of either sex.

Our findings beg the question of how the smArf protein, which lacks N-terminal residues required for p53 activation and is primarily localized to the inner matrix of (maternally inherited) mitochondria [1, 2], would mediate residual tumor suppressive effects in irradiated *smArf* female hosts that are not observed in the *Arf*-null setting. When overexpressed, smArf has been implicated in regulating autophagy [1], although *smArf* mice which encode the p15^smArf^ protein from an engineered cellular *Arf* allele lacking the first methionine codon do not manifest overt defects in mitochondrial respiration in their cultured embryonic fibroblasts [2]. Nonetheless, defects in autophagy affecting other tissues might potentially lead to systemic effects that determine tumor susceptibility [6]. In contradistinction to *BRCA* genes that play general roles in homology-directed DNA repair and whose systemic inactivation predisposes to breast and ovarian cancers [7], *smArf* females are more resistant than their male counterparts to tumor formation in response to IR. In addition, because ALL triggered by Bcr-Abl+, *smArf* donor cells is attenuated in females *versus* males, non-tumor cell autonomous mechanisms might similarly influence disease progression after IR-induced mutagenesis or other oncogenic stress. We are unaware of similar sexual biases in the behavior of other tumor suppressor genes; indeed, the basis for sexual dimorphism stemming from either *BRCA* [7] or *smArf* insufficiency remains a mystery and demands further analysis.
